# Routine lipid profiles in allergic disease: a mini review

**DOI:** 10.3389/falgy.2026.1876509

**Published:** 2026-06-29

**Authors:** Yu Kyoung Hwang

**Affiliations:** Division of Allergy and Clinical Immunology, Department of Internal Medicine, Chungbuk National University Hospital, Cheongju, Republic of Korea

**Keywords:** allergic disease, allergic rhinitis, asthma, atopic dermatitis, high-density lipoprotein, lipid profile

## Abstract

Allergic diseases are traditionally interpreted through epithelial barrier dysfunction, type 2 immune activation, and downstream inflammatory mediators, whereas routine serum lipid measures have usually been considered markers of cardiometabolic risk. However, emerging clinical and mechanistic evidence suggests that high-density lipoprotein (HDL), low-density lipoprotein (LDL), and triglycerides (TG) may provide additional information about systemic inflammatory and metabolic contexts in allergic diseases. This review synthesizes current evidence on routine lipid profiles across asthma, atopic dermatitis, and allergic rhinitis, with emphasis on their clinical associations, disease-specific differences, and potential immune mechanisms. Asthma currently provides the strongest case for clinical relevance, with lipid abnormalities linked to disease burden, metabolic complexity, and heterogeneous clinical outcomes. In atopic dermatitis, circulating lipid measures appear less central than epidermal barrier lipid abnormalities, although they may still reflect selected metabolic or cardiovascular risk contexts. In allergic rhinitis, clinical associations remain inconsistent, but mechanistic studies suggest that lipoprotein function, particularly HDL-related regulation of eosinophils, antigen-presenting cells, and type 2 innate lymphoid cells, may be biologically relevant. Overall, this review presents routine lipid profiles as accessible contextual markers that may help connect systemic metabolism with allergic immune regulation, refine clinical stratification, and generate hypotheses about lipid-immune crosstalk. Future studies integrating routine lipid panels with functional lipoprotein assays, tissue-level immunology, and longitudinal outcome data are needed to determine whether these measures can become useful tools in allergic disease management.

## 1 Introduction

Allergic diseases are usually interpreted through the framework of epithelial barrier dysfunction, type 2 immune activation, and downstream inflammatory mediators (1, 2). Within this framework, lipid biology has most often been discussed in terms of eicosanoids, leukotrienes, and prostaglandins, which are established amplifiers of bronchoconstriction, vascular permeability, pruritus, and leukocyte recruitment (3, 4). By contrast, routine circulating lipid measures such as high-density lipoprotein (HDL), low-density lipoprotein (LDL), and triglycerides (TG) have received much less attention in allergy research and are often treated mainly as indicators of cardiometabolic comorbidity (5, 6).

A growing body of observational work suggests that serum lipid profiles may be linked to asthma prevalence, asthma control, inflammatory burden, or metabolically complex phenotypes (5, 7–9). Smaller and less consistent signals have also been reported in atopic dermatitis (AD) and allergic rhinitis (AR) (10, 11). At the same time, mechanistic studies now indicate that lipoproteins, especially HDL, can directly influence immune-cell behavior through effects on cholesterol handling, lipid raft organization, antigen-presenting cell signaling, eosinophil responses, and type 2 innate lymphoid cell (ILC2) activity (12–14).

In asthma, the literature is developed to support a focused discussion of dyslipidemia as a clinically relevant trait (7). In AD, however, lipid-focused research has been concentrated primarily on epidermal and barrier lipids rather than on circulating HDL, LDL, or TG (15). AR occupies an intermediate position: systemic lipid associations remain inconsistent, but emerging mechanistic work suggests that HDL may influence upper airway type 2 inflammation (14, 16).

**Figure 1:**
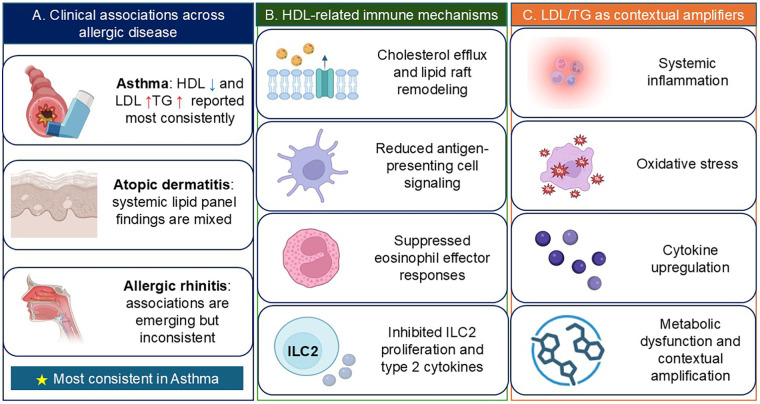
Conceptual framework linking routine lipid panels to immune mechanisms in allergic diseases. **(A)** Clinical patterns across asthma, atopic dermatitis, and allergic rhinitis. Asthma show the most consistent association with lower high-density lipoprotein (HDL) and higher low-density lipoprotein (LDL) or triglyceride (TG) levels, whereas atopic dermatitis and allergic rhinitis show more heterogeneous systemic lipid patterns. **(B)** HDL-related immune mechanisms include cholesterol efflux, lipid raft remodeling, reduced antigen-presenting cell signaling, suppressed eosinophil effector responses, and inhibition of type 2 innate lymphoid cell (ILC2) proliferation and cytokine production. **(C)** LDL and TG-related abnormalities may act as contextual amplifiers through chronic low-grade inflammation, oxidative stress, cytokine upregulation, and metabolic dysfunction.

In this mini review, we examine the current evidence linking HDL, LDL, and TG to allergic diseases, with a primary focus on asthma and with AD and AR considered as comparative conditions. We first summarize the clinical literature on routine lipid panel abnormalities across these diseases. We then discuss mechanistic pathways that may connect circulating lipids to allergic inflammation, emphasizing HDL as a functional lipoprotein rather than cholesterol concentration alone. Finally, we consider how lipid panels may be interpreted clinically, what their major limitations are, and whether they are more likely to support endotyping and risk stratification than diagnosis. The overall conceptual framework is summarized in Figure 1.

## 2 Routine lipid profiles across allergic diseases

### 2.1 Clinical associations across allergic diseases

Among allergic diseases, asthma shows the most consistent clinical association with routine lipid abnormalities (5). Recent syntheses of adult asthma suggest that higher total cholesterol, LDL, and TG levels are often associated with asthma, whereas HDL tends to show an inverse relationship (17). These associations have been reported across cross-sectional, case-control, and cohort designs and are reinforced by the observation that dyslipidemia is linked to worse clinical outcomes in some asthma populations (7). Although the effect sizes are not uniform and many studies remain observational, the overall direction of the evidence supports the view that circulating lipid profiles are not random findings in asthma. Instead, they appear to track with disease burden, metabolic complexity, or specific phenotypic subsets.

This point becomes more interesting when asthma heterogeneity is considered. Dyslipidemia appears particularly relevant in adults with obesity, metabolic syndrome traits or more severe disease, but it may also affect asthma independent of obesity in some cohorts (6, 7). That distinction matters because it suggests serum lipid abnormalities are not simply proxies for body size. Rather, they may reflect a broader immunometabolism state that influences airway inflammation, lung function, or treatment responsiveness (6, 18). For this reason, recent reviews have begun to frame dyslipidemia in asthma as a possible extrapulmonary treatable trait rather than a passive comorbidity (6).

The situation in AD is more complicated. As a disease, AD is strongly linked to lipid biology, but the best-established lipid abnormalities are in the epidermis rather than in serum (15). Barrier lipid disturbances, especially ceramide deficiency and altered free fatty acid compositions, are central to skin permeability and immune activation (19). By contrast, evidence for routine serum lipid panel abnormalities in AD is mixed (10, 20). Some observational studies suggest an association between AD and elevated LDL or cardiovascular risk markers, yet others do not show broad differences in mean total cholesterol, HDL, LDL, or TG after adjustment for confounding factors (21, 22). AR also presents a more heterogeneous pattern. Several studies have reported associations between dyslipidemia and AR severity, quality of life, or symptom burden, and some cohorts have suggested higher prevalence of lipid abnormalities in AR populations (23, 24). However, other studies, including pediatric cohorts, have found no significant correlation between lipid parameters and disease severity indices such as total nasal symptom scores, fractional exhaled nitric oxide (FeNO), or bronchial hyperreactivity (25).

These comparisons suggest that routine lipid panels may serve different roles across allergic diseases. In asthma, they are increasingly plausible as indicators of clinically meaningful heterogeneity. In AD, circulating lipid measures appear less central than epidermal and barrier lipid abnormalities. In AR, they remain hypothesis-generating markers that require closer linkage to symptom burden, lipoprotein function, and type 2 inflammatory mechanisms. Across all three conditions, routine lipid measures are unlikely to function as disease-specific diagnostic biomarkers in isolation. Rather, they may serve as clinically accessible contextual markers of systemic inflammatory and metabolic states that interact with tissue-specific allergic pathways. Table 1 summarizes the disease-specific patterns of HDL, LDL, and triglyceride-related findings across asthma, atopic dermatitis, and allergic rhinitis.

**Table 1:** Comparative summary of HDL, LDL, and triglyceride-related findings across allergic diseases.

Disease	Typical lipid panel pattern	Strength of clinical association	Mechanistic relevance	Most likely interpretation	Translational implication
Asthma	Lower HDL and higher LDL/TG are reported most consistently, although not uniformly across all cohorts	Moderate to relatively strong	Most developed, with evidence linking HDL to immune regulation and LDL/TG to systemic inflammation, oxidative stress, and metabolic dysfunction	Routine lipid abnormalities may reflect a metabolically complex asthma phenotype rather than asthma alone	Most promising setting for endotyping, risk stratification, and exploration of dyslipidemia as a treatable trait
Atopic dermatitis	Serum lipid panel findings are mixed; some studies suggest higher LDL, but broad HDL/LDL/TG differences are inconsistent	Weak to moderate	More limited for serum lipid panels; strongest lipid biology remains at the level of epidermal/barrier lipids rather than circulating lipoproteins	Routine lipid panels likely provide only an indirect or secondary signal compared with local skin lipid abnormalities	Limited role as a stand-alone marker; may be useful mainly in broader metabolic or cardiovascular risk assessment
Allergic rhinitis	Associations with dyslipidemia, symptom severity, or quality of life have been reported, but findings are inconsistent	Emerging/limited	Selective but interesting, especially for HDL, which has been linked to ILC2 suppression and reduced type 2 cytokine production	Lipid panels may capture systemic inflammatory context, but current evidence is not sufficient for robust disease-level conclusions	Potential future role in mechanistically informed phenotyping, but currently hypothesis generating

### 2.2 Lipid immune mechanisms

Among routine lipid measures, HDL is the most mechanistically compelling because it is not merely a cholesterol carrier but a functionally complex lipoprotein with anti-inflammatory, antioxidative, and immunomodulatory properties (26). Experimental and translational studies show that HDL can promote cholesterol efflux from cells and alter the cholesterol content of membrane microdomains known as lipid rafts (27). These rafts are enriched in cholesterol and sphingolipids and are essential for clustering receptors involved in antigen presentation, Fc receptor signaling, and cytokine responses (28, 29). By depleting cholesterol from these domains, HDL can disrupt raft-dependent immune signaling and thereby lower inflammatory activation in specific contexts.

This concept helps explain how HDL may influence allergic inflammation beyond cardiometabolic risk. In antigen-presenting cells, HDL and HDL-associated apolipoproteins can reduce the capacity to stimulate T-cell activation by modifying membrane organization (27). HDL-associated lipids and apolipoproteins have also been shown to suppress eosinophil effector responses, including shape change and migration (30, 31). Importantly, HDL is not a single uniform entity. Its composition and function differ across disease states (32, 33). In allergic and inflammatory disorders, HDL may lose some of its anti-inflammatory properties or acquire altered effects depending on its apolipoprotein cargo, phospholipid composition, and antioxidative capacity (13).

Experimental work has shown that HDL, particularly its major apolipoprotein ApoA-I, can inhibit proliferation and type 2 cytokine production by ILC2s, partly through downregulation of GATA3- and ROR-related pathways (34). Because ILC2s are key drivers of type 2 airway inflammation, this finding provides a direct link between a circulating lipoprotein and a central allergic effector pathway (35). Together with observations that HDL may suppress eosinophil activity and modulate antigen-presenting cell behavior, these data position HDL as a plausible bridge between systemic lipid metabolism and allergic immune regulation.

LDL and TG are mechanistically less specific but still biologically relevant. Elevated LDL and TG-rich dyslipidemia are associated with low-grade systemic inflammation, oxidative stress, endothelial activation, and altered cytokine production (36, 37). In asthma, these processes may contribute to airway inflammation indirectly through circulating inflammatory mediators, altered macrophage polarization, changes in pulmonary sterol handling, and interaction with obesity-related metabolic dysfunction (38, 39). Diet-induced hypercholesterolemia can activate inflammatory pathways such as Toll-like receptor and NF-kappaB signaling in animal models, and dyslipidemia has been linked to reactive oxygen species generation, which may exacerbate airway injury and immune activation (36, 40). Recent reviews also highlight shared genetic and lipid-metabolic pathways, including ORMDL3-sphingolipid biology, as potential connectors between lipid dysregulation and asthma susceptibility (41, 42).

### 2.3 Disease-specific interpretation

Asthma currently offers the strongest case for integrating routine lipid panels into allergic disease interpretation (6, 7, 17). The clinical association data are the most extensive, the mechanistic pathways are the most developed, and the concept of dyslipidemia as a treatable trait is most clearly established in asthma (38). In asthma, low HDL and elevated LDL or TG may identify a metabolically complex inflammatory phenotype in which airway disease coexists with systemic low-grade inflammation, oxidative stress, and altered lipid handling (6, 7, 38). Such patients may differ not only in cardiometabolic risk but also in inflammatory tone, disease severity, exacerbation risk, and possibly treatment responsiveness.

In AD, by contrast, the key message is not that routine serum lipid panels are irrelevant, but that they are secondary to more established tissue-level lipid biology (15). Barrier lipid abnormalities remain central to AD pathogenesis, whereas circulating HDL, LDL, and TG provide a weaker and less consistent signal (10, 19). Some studies suggest associations with LDL-C or cardiovascular risk profiles, but these observations do not yet support a robust model in which routine lipid panels explain AD heterogeneity (21).

AR occupies an intermediate position between asthma and AD. Epidemiologic evidence is not strong enough to support definitive panel-based conclusions, yet the HDL-ILC2 literature suggests that mechanistic insight may emerge before consistent clinical biomarkers do (23, 25, 34). A disease may show weak or inconsistent serum lipid associations while still offering informative mechanistic pathways, particularly when the relevant biology concerns lipoprotein function rather than total circulating concentration (13).

## 3 Discussion

From a clinical perspective, the strongest current use case for routine lipid panels in allergy is not diagnosis but clinical stratification. Because HDL, LDL, and TG are commonly measured for cardiometabolic risk assessment, they are attractive markers for identifying metabolically complex allergic phenotypes without requiring disease-specific assays. In asthma, this could help distinguish patients whose disease burden is shaped partly by systemic inflammation and dyslipidemia (6, 7). In AD and AR, routine lipid panels may be less informative at present but may still contribute to broader clinical phenotyping in selected settings. Among lipid-derived indices, elevated TG/HDL-C ratios have also been associated with asthma burden and airway obstruction, including in pediatric asthma populations (43).

The biological rationale for this stratification differs across lipid measures. Among these markers, HDL has the clearest immunologic relevance because it acts as a functional lipoprotein capable of modulating membrane signaling, antigen presentation, eosinophil behavior, and ILC2-driven inflammation (13, 27, 33, 34). LDL and TG appear more closely linked to broader systemic inflammatory and metabolic states, which may still be relevant in metabolically complex allergic disease (36).

Age may further influence the interpretation of lipid profiles in allergic diseases. Pediatric studies have reported mixed but clinically relevant associations between lipid parameters and asthma-related outcomes, including airway obstruction and bronchial hyperresponsiveness (5, 43). Lipid profiles in children may also be influenced by growth and obesity-related metabolic factors (44, 45). In adults, lipid abnormalities are more often linked to metabolic syndrome and cardiometabolic risk, with the strongest evidence observed in asthma (6, 7, 17). These differences suggest that lipid profiles should be interpreted in relation to age, metabolic status, and disease phenotype.

The main limitation of the current literature is that most studies are observational and vulnerable to major confounding (17, 20). Obesity, body mass index (BMI), diet, physical activity, lipid-lowering therapy, corticosteroid exposure, diabetes risk, and socioeconomic factors can all influence both lipid levels and allergic outcomes (6, 46). In addition, lipid values are often measured at a single time point, which obscures whether they represent stable traits, disease consequences, or treatment effects. Mendelian randomization and longitudinal cohorts will therefore be essential for clarifying which signals are causal, which are merely correlative, and which simply reflect shared background risks (20, 47).

Future studies should move beyond lipid concentration alone. HDL function, lipoprotein composition, oxidized lipid species, and paired tissue-blood comparisons are likely to be more informative than routine panels alone (13, 32, 48). It will also be important to define whether lipid abnormalities remain stable across exacerbations and remission, whether they change in response to biologic therapies, and whether they predict outcomes beyond what can already be inferred from obesity or metabolic syndrome (7, 49). A major advance would be the integration of routine lipid panels with eosinophil counts, FeNO, IgE, or transcriptomic endotypes to test whether combined models outperform isolated biomarkers (50, 51). A key future question is whether specific lipid patterns identify endotypes with distinct immune mechanisms, prognosis, or therapeutic needs.

Taken together, the value of routine lipid measures may lie in capturing systemic context, refining endotypes, and prompting mechanistic questions about lipid-immune crosstalk. A focused research agenda that integrates clinical lipid panels with functional lipoprotein assays and tissue-level immunology will be necessary to determine whether these common laboratory measures can become meaningful tools in allergic disease stratification and management.
